# LED-based, real-time, hyperspectral imaging device

**DOI:** 10.1117/1.JMI.12.3.035002

**Published:** 2025-06-12

**Authors:** Naeeme Modir, Maysam Shahedi, James Dormer, Ling Ma, Baowei Fei

**Affiliations:** a University of Texas at Dallas, Department of Bioengineering, Richardson, Texas, United States; b University of Texas at Dallas, Center for Imaging and Surgical Innovation, Richardson, Texas, United States; c University of Texas Southwestern Medical Center, Department of Radiology, Richardson, Texas, United States

**Keywords:** hyperspectral imaging, multispectral imaging, endoscope, FPGA, LED array, gastrointestinal cancer

## Abstract

**Purpose:**

This study demonstrates the feasibility of using an LED array for hyperspectral imaging (HSI). The prototype validates the concept and provides insights into the design of future HSI applications. Our goal is to design, develop, and test a real-time, LED-based HSI prototype as a proof-of-principle device for *in situ* hyperspectral imaging using LEDs.

**Approach:**

A prototype was designed based on a multiwavelength LED array and a monochrome camera and was tested to investigate the properties of the LED-based HSI. The LED array consisted of 18 LEDs in 18 different wavelengths from 405 nm to 910 nm. The performance of the imaging system was evaluated on different normal and cancerous *ex vivo* tissues. The impact of imaging conditions on the HSI quality was investigated. The LED-based HSI device was compared with a reference hyperspectral camera system.

**Results:**

The hyperspectral signatures of different imaging targets were acquired using our prototype HSI device, which are comparable to the data obtained using the reference HSI system.

**Conclusions:**

The feasibility of employing a spectral LED array as the illumination source for high-speed and high-quality HSI has been demonstrated. The use of LEDs for HSI can open the door to numerous applications in endoscopic, laparoscopic, and handheld HSI devices.

## 1 Introduction

Gastrointestinal (GI) cancers include the esophagus, pancreas, stomach, colon, rectum, anus, liver, biliary system, and small intestine cancers. The statistics reported in the literature show that in 2024 in the United States, digestive system cancers are among the most common cancers, with more than 353,000 and 174,000 estimated annual incidence and mortality rates, respectively.^1^ Endoscopy is a minimally invasive optical imaging technique used to visualize internal sites of the body through natural openings. As one of the standard procedures in the last two decades, GI endoscopy has played a critical role in diagnosing GI cancers. GI cancer screening by endoscopy has demonstrated a strong impact on localized cancer prognosis and diagnosis rate.^2^ In the 2000s, the widespread use of colonoscopy contributed to a rapid decline in the incidence of colorectal cancer.^1^ However, conventional GI endoscopy has limitations. For example, ∼8% to 11% of tumors are missed due to the lack of visibility during upper GI endoscopy.^3^^,^^4^

One potential solution to improve the sensitivity of endoscopic examinations is the use of hyperspectral imaging techniques. Hyperspectral imaging is an optical imaging technique utilized in biomedical imaging to detect and diagnose diseases such as cancer, cardiac disease, dermatological conditions, and retinal disorders.^5^^–^^8^ It has demonstrated its strength in visualizing tumors that are invisible to conventional color cameras used in conventional endoscopy.^5^^,^^8^ In conventional endoscopic imaging, a camera captures color images using wideband red, green, and blue (RGB) channels. However, an RGB system has limited spectral resolution and wide bandwidth, which can make it challenging to differentiate all abnormal tissues from benign ones. By contrast, a hyperspectral imaging system captures numerous images across various discrete, narrowband wavelength channels, including wavelengths beyond the visible spectrum. This allows it to track biochemical changes in cancerous tissue, which exhibit distinct signatures under spectral imaging, making it an effective solution to address the limitations of conventional endoscopy in cancer detection.^5^^,^^9^^–^^12^ Moreover, as the visual properties of resected tissues change during pathological examinations,^13^ *in vivo* noninvasive tissue screening techniques could be beneficial prior to the resection procedure. Hyperspectral imaging can facilitate rapid *in vivo* assessment of tissues over a larger area before resection. This approach could accelerate the diagnosis process and reduce costs by minimizing unnecessary tissue resections and pathological tests.

There are four typical approaches to hyperspectral imaging scanning: point scanning, line scanning (also known as pushbroom), wavelength scanning, and snapshot.^14^ Point and line scanning approaches are slow but can provide high spectral resolutions. The snapshot approach, on the other hand, is fast but usually limited in spectral resolution.^15^ Wavelength scanning is an imaging method that offers relatively high speed along with high spectral and spatial resolutions. For endoscopic imaging, a real-time or near-real-time imaging rate is essential. In hyperspectral systems, a frame rate of ∼20 hypercubes per second (hps) or higher is considered real-time.^15^ However, some hyperspectral endoscopic imaging systems are also proposed as real time or near real time with imaging rates of <10  hps.^16^^,^^17^ To achieve real-time hyperspectral imaging in endoscopy, wavelength scanning and snapshot imaging methods are generally more efficient than point or line scanning. However, the imaging mechanism of snapshot hyperspectral imaging poses challenges for high-quality endoscopic imaging.^16^

Several multi- and hyperspectral endoscopes have been presented in the literature. For example, Yoon et al.^18^ presented a freehand line scanning hyperspectral endoscopic imaging system with high spectral and spatial resolutions. Their system can acquire hyperspectral data at an imaging rate of over 20 hps within the 450 to 710 nm spectrum, although hypercube reconstruction requires up to 50 s of post-processing. A snapshot hyperspectral endoscope with an imaging rate of ∼6  hps has been described in Ref. 15. This system captured small hypercubes through a set of 100 optical fiberlets, each with a size of 10  pixels×10  pixels×756 wavelengths. The field of view (FOV) of this system is 1.1  mm×1.3  mm and cannot be used for large-scale visualization. Kester et al.^16^ presented a near-real-time (∼5  hps) snapshot hyperspectral endoscope with a hypercube size of 350×350×48, a spectral range of 450 to 650 nm, and a 10 mm field of view. In their system, images were transmitted through a fiber bundle to an image mapper, which divided them into a set of image rows. Each row was then mapped to a dedicated imaging element, and the hyperspectral images of each row were combined to form the hypercube. The image mapping approach required an image mapper, an array of prisms, an array of lenses, and an array of image sensors, as well as precise fabrication and implementation. This system introduced periodic intensity artifacts due to mapper fabrication defects. Han et al.^13^ proposed a wavelength scanning multispectral endoscope for colorectal cancer detection, utilizing a mechanical wheel to filter white light and generate different wavelengths. Optical fibers were used to transfer light into the body. Due to the mechanical filtering system, the number of spectral bands was limited to five for real-time imaging, and the selected wavelengths were customized to visualize colorectal abnormalities. Hohmann et al.^19^ presented an *in vivo* multispectral endoscope with six wavelengths spanning 400 to 650 nm. The imaging time for their system was ∼0.45  s per datacube for image acquisition, with an additional 2 s for pre- and post-processing for image reconstruction.

For hyperspectral imaging, it is common to use tens to hundreds of spectral bands. However, in clinical applications such as hyperspectral endoscopic imaging, a limited number of spectral bands within a specific range can sometimes be sufficient for accurate diagnosis of abnormalities. For instance, Liu et al.^9^ demonstrated that gastric cancer can be diagnosed using near-infrared (NIR) hyperspectral imaging. They identified six optimal wavelengths for diagnosing gastric cancer: 975, 1075, 1215, 1275, 1390, and 1450 nm. In addition, Akbari et al.^10^ proposed wavelength ranges of 1226 to 1250 nm and 1288 to 1370 nm for distinguishing between cancerous and noncancerous gastric tissues. Raj et al.^20^ found that violet light at a wavelength of 420 nm could enhance the contrast between blood vessels and surrounding mucosa, making it useful for detecting angiogenesis, an important early indicator of cancer growth. Qiu et al.^21^ utilized a spectral range of 600 to 800 nm for detecting precancerous conditions in the esophagus. Mitsui et al.^22^ conducted an *ex vivo* study to show that using NIR hyperspectral imaging could be helpful for identifying the extent of gastric cancer that cannot be detected during conventional endoscopy. Thus, using a wavelength scanning approach with a limited number of wavelengths within a clinically relevant range can help address the challenges of limited imaging rate and spatial resolution. This approach also reduces the post-processing computational time required for image reconstruction compared with other hyperspectral imaging methods. However, wavelength scanning methods that use rotating filter wheels, liquid crystal tunable filters, or acousto-optic tunable filters can be relatively slow, which can lower the imaging frame rate, particularly when multiple wavelengths are used for imaging.^14^

Due to the broad range of LED wavelengths and their high switching speeds, using multispectral *in situ* LED-based illumination can enhance the imaging rate during wavelength scanning while addressing issues associated with fiber bundles. For example, Dung et al.^23^ designed and implemented a narrowband capsule endoscope that utilizes white light and a single LED with a central wavelength of 430 nm. This system achieved an imaging rate of 2 hps. Iakovidis et al.^24^ investigated the feasibility of a multispectral wireless capsule endoscope (WCE). They proposed a field-programmable gate array (FPGA)-based implementation of an automatic abnormality detection algorithm embedded in the capsule, utilizing image feature extraction and machine learning–based classification methods. The spectral resolution and imaging rate of their endoscope are not specified. Shrestha and Hardeberg^25^ used a simulation study to examine the influence of noise and the number of LED channels in a multispectral imaging system using multiwavelength LED illumination. They assessed both RGB and monochrome cameras for capturing spectral images with a multi-channel LED light source. The study explored how camera type and the number of wavelengths in the light source affected spectral imaging quality. Their findings showed that increasing the number of LED channels with a monochrome camera improves image quality, whereas using an RGB camera for spectral imaging provides faster imaging at the expense of reduced quality. These findings are based on simulations and have not yet been validated through experimental testing. There are also studies published on using LED-based hyperspectral laparoscopy. Pfahl et al.^26^ developed an LED-based wavelength scanning laparoscopic imaging system that utilized 14 narrowband LEDs, each at a different wavelength ranging from 405 to 960 nm. Light was delivered to the imaging site via a light guide, and spectral images were captured using a high-resolution RGB camera with an imaging rate of 60 frames per second (fps). The final spectral imaging rate of their system was less than two hps.

In this study, we propose using an LED-based wavelength scanning hyperspectral imaging approach to improve the image capturing rate to over 10 hps without compromising spatial resolution. Using an *in situ* hyperspectral light source, we avoid fiber optics in our design to increase the mobility of the endoscope catheter and mitigate the complexity of its mechanical design. In contrast to the hyperspectral imaging approaches in which the light is delivered with a fiber bundle inserted through the working channel of the endoscope, our design enables leaving the working channel of the endoscope catheter available for any intra-imaging procedures under hyperspectral imaging guidance. When a spectral LED-based illumination is used in a wavelength-scanning hyperspectral imaging (HSI) approach, the imaging target tissue is exposed to each wavelength for a very short time, and therefore, the target tissue is less affected by the illumination (e.g., photochemical damage or an increase in temperature) compared with a broad range white illumination usually used in hyperspectral imaging. Moreover, it helps to customize the combination of spectral bands used for imaging based on clinical applications and improve the diagnostic characteristics of the hyperspectral endoscopic imaging as well as its imaging rate. Another advantage of using an LED-based light source in our design is the ability to dynamically adjust illumination intensities based on the distance to the target. Real-time control of illumination intensity is critical in endoscopy, as excessive light can cause sensor saturation, whereas insufficient light may lead to noisy images and underexposure artifacts.^27^ The control module can be designed to control the illumination of each LED by adjusting the drive current supplied to it. Spatial resolution is another limitation of previously presented hyperspectral endoscopes.^15^ The proposed method can facilitate high-resolution imaging at a high imaging rate. It also enables controlling spatial resolution whenever a higher imaging rate is needed. Another limiting factor in endoscopic imaging devices is size. Using micro-LEDs with microscopic footprints smaller than 400  μm×400  μm enables the accommodation of tens of LEDs at the tip of a clinical endoscopic catheter. The LEDs are mounted on a PCB surrounding the camera.

In this paper, our main contributions are: (1) the design and schematic of a hyperspectral endoscope incorporating micro-LEDs at the catheter tip and (2) a feasibility study evaluating the application of an LED-based illumination source for endoscopic imaging, validated through large-scale prototyping. In Sec. 2.1, we briefly explained our proposed design for a real-size hyperspectral endoscopic imaging system using a micro-LED-based on-tip illumination source. We also explained a large-scale prototyped HSI system designed to investigate the feasibility of using spectral LED-based illumination for fast and high-quality HSI in Sec. 2.2. We designed some experiments to assess the imaging performance of our prototype and compared it with the performance of a reference HSI camera. In Sec. 3, we presented our results, and in Sec. 4, we discussed our observations and explained the advantages and limitations of our design. Finally, we concluded our study in Sec. 5.

## 2 Materials and Methods

### 2.1 Proposed Hyperspectral Imaging Endoscope Design

**Fig. 1:**
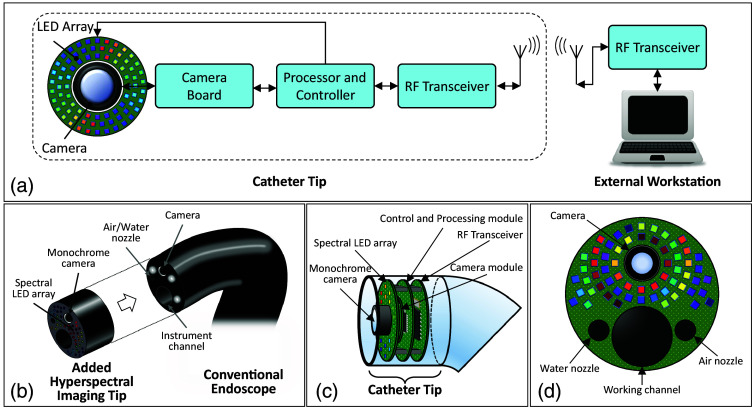
Schematic block diagrams of the real-size hyperspectral endoscopic imaging system: (a) the whole endoscopic imaging system; (b) the attachment of the proposed hyperspectral endoscopic imaging system to a clinical GI endoscope; (c) the internal parts of the proposed catheter tip; and (d) the LED array PCB.

We proposed a miniature hyperspectral imaging system. The concept and design of the device was described earlier.^28^ Figure 1(a) shows the schematic block diagram of the proposed imaging system. We designed our imaging system based on *in situ* LED illumination and a monochrome camera [Fig. 1(c)]. The imaging system consists of two main parts: an internal catheter tip for image acquisition [Fig. 1(b)] and an external workstation for further processing of the data. The catheter tip is composed of imaging, control and processing, and transceiver modules. The system could communicate wirelessly with an external workstation for transferring imaging data and receiving control commands. Therefore, the proposed hyperspectral endoscope can be attached to the tip of a clinical endoscope catheter, as shown in Fig. 1(b). The light source consists of a multiwavelength LED array mounted on a PCB, which surrounds the monochrome camera. Figure 1(d) shows an approximate arrangement of the LED array at the tip of a clinical GI endoscope. The illumination of the LEDs is controlled by the control and processing module. This module is also responsible for controlling the system’s operational modes and the camera. For a real-size endoscope, an ASIC design for control and processing can miniaturize the design. The wireless transceiver sends the imaging data to the workstation outside the body for further processing. Besides, it receives commands from the workstation for setting the control parameters of the endoscope. The control and processing module, along with the digital baseband module and analog frontend, can be integrated as a system-on-chip (SoC) for further miniaturizing the system. The data processing, which consists of the required soft calibration, image enhancement, image channel integration, and real-time image classification, can be done on the workstation. The maximum frame rate of the camera and the number of scanned wavelengths are two factors that limit the rate of hyperspectral imaging.

### 2.2 Proof-of-Principle Prototype Development

**Fig. 2:**
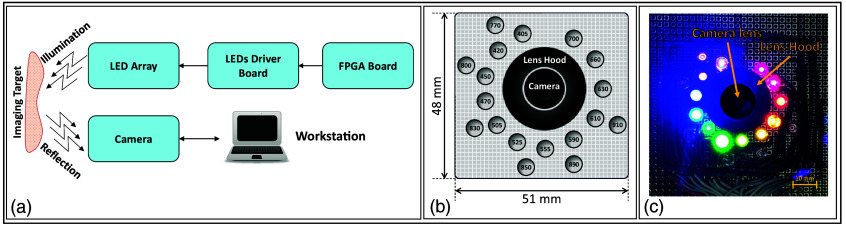
(a) Schematic block diagram of the prototyped hyperspectral imaging system. (b) The schematic diagram of the LED array. Each circle shows the location of one through-hole LED, and the number inside each circle shows the wavelength of the LED in nm unit. (c) A snapshot of the LED array when all the LEDs are on for display only (the light of IR LEDs was not captured by the camera used for taking this photo).

To evaluate the image quality of the proposed hyperspectral endoscopic imaging system, we designed, developed, and tested a large-scale prototype of the proposed imaging system. Figure 2(a) shows the schematic block diagram of the prototype imaging system. The prototype consisted of a spectral array of through-hole LEDs mounted on a board surrounding a micro digital monochrome camera [see Figs. 2(b) and 2(c)]. The imaging system was placed inside a dark box to block the environmental light and simulate the endoscopy lighting condition. The illumination of the LED array was controlled by an FPGA and a driver board. The LED driver board included a set of precision potentiometers designed to fine-tune the driving current supplied to each LED. This adjustment mechanism enabled precise control over the brightness levels of the individual LEDs. Below, we provided a detailed explanation of each component of the prototype.

#### 2.2.1 Imaging module

**Fig. 3:**
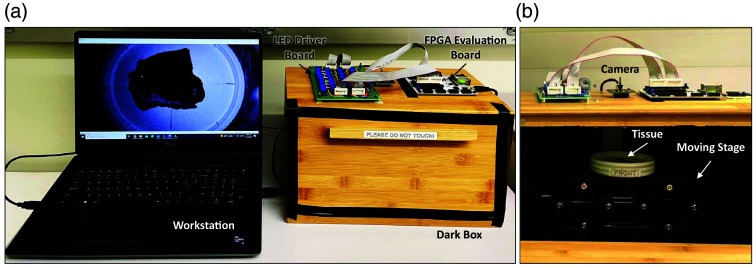
(a) Experiment setup where the FPGA and LED driver boards control the LEDs inside the dark box and the laptop workstation connected with the boards for the image acquisitions. (b) Inside the dark box, the tissue was placed on a stage that can change its height, and the LEDs were controlled to perform the hyperspectral scanning.

The imaging module consisted of an LED array and a monochrome camera installed inside a dark box. An adjustable imaging target stage was placed in the dark box right under the camera lens to control the distance of the imaging target from the imaging module. As shown in Fig. 2(c), the camera lens was optically shielded by a lens hood to avoid any direct illumination from LEDs. Figure 3 shows the setup we used in this study.

##### LED array

The LED array was used as a multiwavelength light source. The LEDs were turned on and off consecutively to provide light with different wavelengths. In our prototype, we used 18 through-hole LEDs at 18 different wavelengths from Marubeni Corporation (Tokyo, Japan). The wavelengths of the LEDs ranged from the UV and visible light spectrum (405 to 700 nm) to the NIR and IR (700 to 910 nm). Table 1 shows the specifications of each LED used in this study. Figure 2(b) shows the schematic diagram of the LED array and the location of each LED with respect to the camera lens. Figure 2(c) shows a photo of the LED array when all the LEDs were on for display purposes only. The lens hood protected the lens from receiving light directly from LEDs.

**Table 1:** Specifications of the LEDs used in the experiments.

Wavelength (nm)	Half width (nm)	Viewing angle (deg)
405	19	102
420	16	26
450	20	40
470	25	80
505	30	86
525	30	18
555	25	40
590	13	64
610	15	30
630	15	60
660	18	80
700	21	80
770	26	14
800	29	88
830	35	14
850	40	26
890	75	40
910	47	30

##### Camera

For the imaging module, we used the UC10MPC_L36 camera manufactured by Spinel (Orange County, California, United States). This was a CMOS monochrome camera with a 3.6 mm lens and an FOV of 90 deg. The image sensor size was 0.25 inches with 1280×720  pixels. The physical pixel size was 3  μm×3  μm. The camera has no optical filter. The maximum imaging frame rate of the camera was 120 fps. At the adjusted distance between the camera lens and the imaging target in this study, the FOV was ∼91  mm×51  mm, corresponding to a physical pixel size of 71  μm.

#### 2.2.2 Control module

In this study, we used the Xilinx Artix-7 FPGA on the Arty-7 evaluation board from Digilent (San Jose, California, United States) as the control module for the prototyped system. The FPGA sequentially turned on and off the LEDs during the imaging process by providing 18 waveforms to drive the 18 LEDs. Each waveform consisted of a short, square-shaped pulse with a pulse width of PW. LEDs were activated one at a time while keeping the others off. A delay (DT) was introduced after turning off an LED and before activating the next one. This sequential activation pattern was repeated continuously. PW and DT were controlled by the control module based on the requirements of imaging experiments such as imaging rate and quality.

### 2.3 Evaluation of the prototyped hyperspectral imaging system

To evaluate the performance of our hyperspectral imaging prototype, we used a customized hyperspectral camera (SNAPSCAN VNIR, IMEC, Leuven, Belgium) as a reference for this study. Before each imaging session, we calibrated our HSI system, as described in Sec. 2.3.1. We also calibrated the reference HSI camera based on its protocol. The reference camera had an imaging size of 2048×2048  pixels. At the adjusted distance between the camera and the imaging target in this study, the FOV was ∼102  mm×102  mm, corresponding to a physical pixel size of 50  μm×50  μm. After capturing the images, for a better comparison between our system and the reference one, we manually registered the images of the reference HSI camera to the corresponding images of our system using a rigid transformation (see Sec. 2.3.2). Image registration enabled the extraction of hyperspectral signatures of each imaging target from the same regions of interest (ROIs) on both hypercubes. In this study, we used in-house software developed in MATLAB (R2024a, MathWorks, Natick, Massachusetts, United States) to perform manual rigid image registrations.

#### 2.3.1 Calibration

The intensity and distribution of the LEDs’ light, as well as the camera sensor’s sensitivity to different wavelengths, could vary. Therefore, a calibration of the image intensity is required at the beginning of each imaging session to adjust the intensity of the spectral images. The calibration process compensates for both the spatial and spectral inhomogeneity of the LED array illumination and the spatial inhomogeneity of camera sensor sensitivity. The calibration involves two steps: first, capturing a dark current image with no light and, second, capturing images of the reflected intensities from a white reference while each LED illuminates the scene individually. For Λ spectral channels (here Λ=18), the calibrated spectral image, I^(x,y,λ), after canceling out the intra-channel inhomogeneity of the illumination distribution and inter-channel illumination inconsistency, is given as

I^(x,y,λ)=I(x,y,λ)−ID(x,y)IW(x,y,λ)−ID(x,y),λ∈{1,2,3,…,Λ},

where for each pixel at (x,y) coordinate, I(x,y,λ) is the intensity (i.e., reflectance) of the pixel for the λth band before calibration, $I_{D}(x,y)$ is the intensity of the dark current image captured at (x,y) when all the LEDs are off and $I_{W}(x,y,\lambda )$ is the intensity of the λth band at (x,y) when a white reference is used as the imaging target.

In this study, we use two LabSphere Spectralon reflectance standards (Labsphere Inc., North Sutton, NH, USA) as white references for calibration purposes: 10×10 inches (SRT-99-100, hereafter called White Reference #1) and 5×5 inches (SRT-99-050, hereafter called White Reference #2). White Reference #2 was used when White Reference #1 could not be used due to the physical limitations of the prototype dark box the. For example, for testing the impact of depth of field on the calibration process.

#### 2.3.2 Image alignment, segmentation, and normalization

When comparing the performance of our system to a reference hyperspectral imaging system, it is necessary to account for differences in imaging orientation and magnification between the systems. To achieve this, we manually register the hyperspectral images captured by the reference system with those captured by our LED-based system. The image registration process involves a rigid transformation, including translation, rotation, and scaling, to align the images accurately. Next, we apply an automatic image segmentation algorithm based on histogram analysis to remove the background and glare artifacts from each pair of hypercubes. In this study, the imaging targets are placed on a white dish, causing the background to appear as a hyperintense region in the images. The glare artifacts, which also appear as hyperintense regions, can be effectively segmented along with the background using a histogram thresholding approach.^29^ To ensure consistent processing of the same regions on the target tissue for both the prototype and reference imaging system, we combine the two segmentation masks obtained from each system by identifying their overlap. This results in a single segmentation mask that defines the common ROI in both the prototype and reference hypercubes. We then extract the spectral signatures of the tissues from the defined ROI. To have a better comparison between the two spectral imaging systems, we applied the following normalization process to the spectral signatures

S^(λ)=S(λ)−SminSmax−Smin,

where S(λ) is the original value of the spectral signature at the λth wavelength, and $S_{\min}$ and $S_{\max}$ are the minimum and maximum values of the original signature across all the wavelengths, respectively. S^(λ) is the normalized spectral signature value at wavelength λ.

### 2.4 Evaluation the performance of hyperspectral imaging using LED-based illumination

We conducted a set of experiments on *ex vivo* tissues to evaluate the performance of the prototyped LED-based HSI system. We captured images of several normal and cancerous tissues using our hyperspectral imaging prototype. We also used a customized HSI camera as a reference and imaged the same set of tissues. The reference camera used in these experiments covered 150 wavelengths from 470 to 900 nm with a spectral resolution of ∼3  nm. We compared the acquired hypercubes of our system to the corresponding reference hypercubes. For the *ex vivo* imaging experiments, we used different normal tissues resected from different animal models, including chickens (thigh muscle tissues), sheep (brain, heart, liver, and kidney tissues), and mice (heart, liver, and kidney tissues). As we reported in one of our previous studies, tumor tissues resected from the kidneys of four mice were used for our *ex vivo* imaging tests.^30^ The study was approved by the research ethics board of our institution. Before starting the experiments, we calibrated both imaging systems using white reference #1. For capturing images from each tissue, we put it on the imaging stage inside the dark box and sealed the box to block any environmental light. Then, we turned on the first LED for 2 s, captured the images, and then turned off the LED (i.e., PW = 2 s). The next LED was turned on with a delay of 2 s (DT = 2 s). We turned on and off the LEDs one by one and captured the images of the different spectral channels. We measured the signal-to-noise (SNR) level using the average reflectance of the image ($\mu_{\mathrm{ROI}}$) divided by its standard deviation ($\sigma_{\mathrm{ROI}}$)

$${\mathrm{SNR}}_{\mathrm{dB}}=20\;\log_{10}(\frac{\mu_{\mathrm{ROI}}}{\sigma_{\mathrm{ROI}}}).$$

**Fig. 4:**
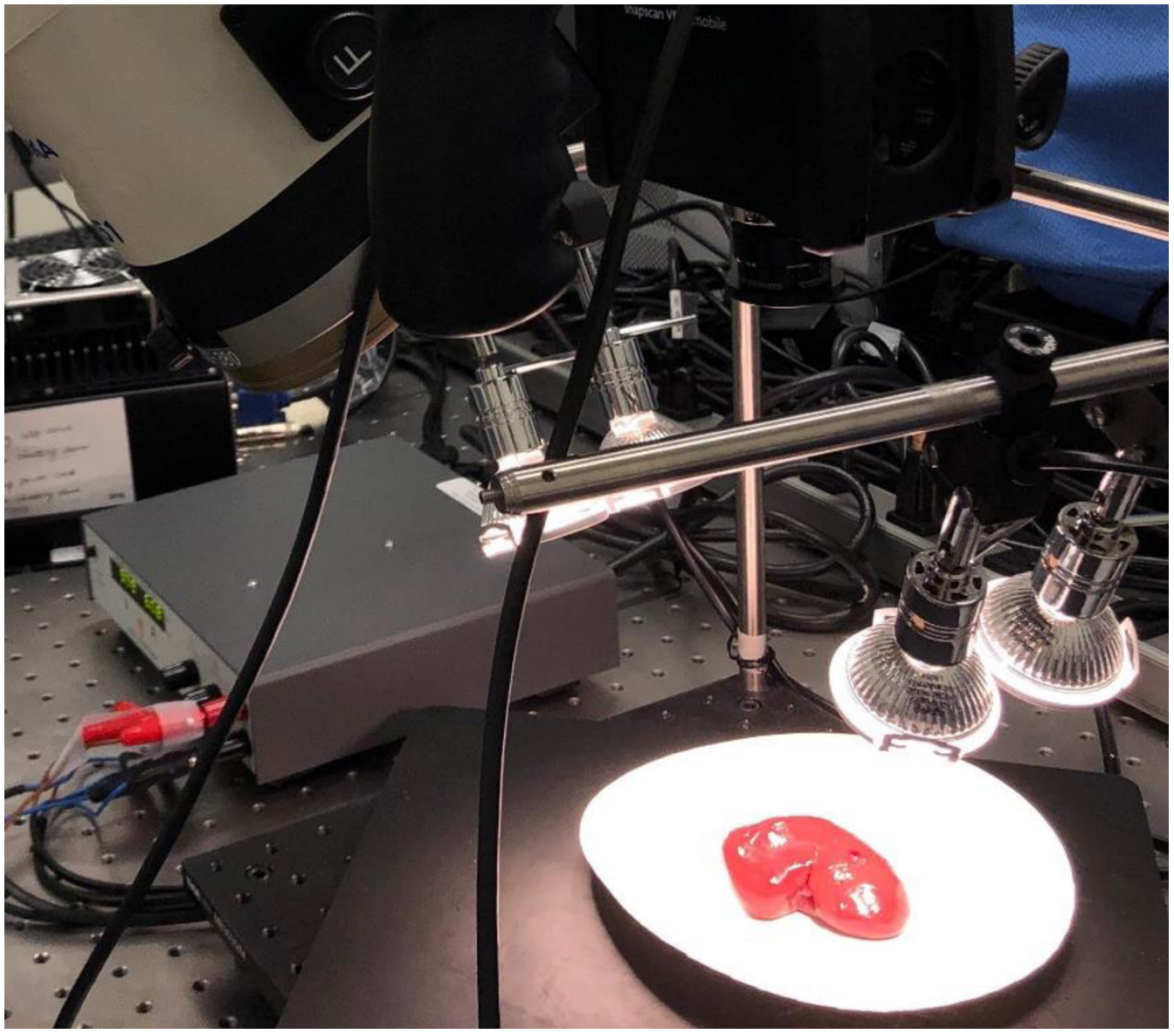
Reference imaging setup using a customized HSI camera and halogen lights. The imaging target tissues are sheep kidneys (renal capsules) ∼85  mm in length.

Figure 3 shows the imaging setup for our prototype, and Fig. 4 shows the imaging setup for the reference HSI camera. The post-imaging pairwise comparison between the hyperspectral signatures was done after normalization of the signatures.

## 3 Results

### 3.1 Calibration

**Fig. 5:**
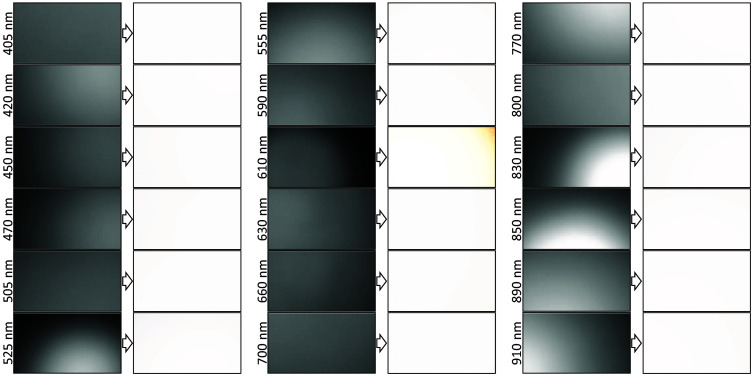
Spectral images of the white reference #1 before (left) and after calibration (right) for the 18 spectral bands. The illumination artifacts after calibration have been highlighted in orange color.

**Fig. 6:**
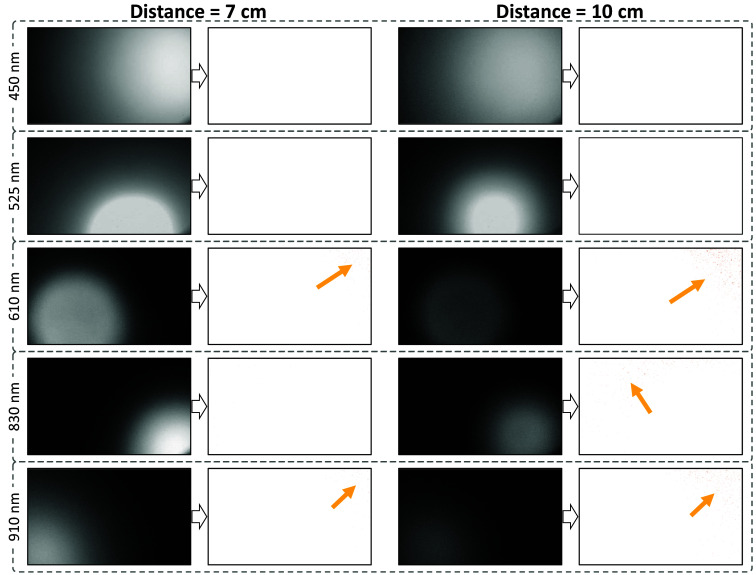
Spectral images of the white reference #2 imaged at two different distances [7 cm (left section) and 10 cm (right section)] from the illumination source and camera, before (left images) and after calibration (right images) for five sample spectral bands. The illumination artifacts after calibration have been shown with orange arrows.

Figure 5 shows the images of all 18 spectral channels from white reference #1 before and after applying the calibration process. As shown in Fig. 6, we also imaged white reference #2 from two different depths of field (DOFs) (7 and 10 cm) for the calibration process to compare the impact of DOF on the calibration process. For exposure times ranging from 20 to 200 ms, the SNR remained above 15 dB for all bands except at 450, 470, and 525 nm, where the SNR values exceeded 10 dB.

### 3.2 Evaluating the Performance of Hyperspectral Imaging Using LED-Based Illumination

**Fig. 7:**
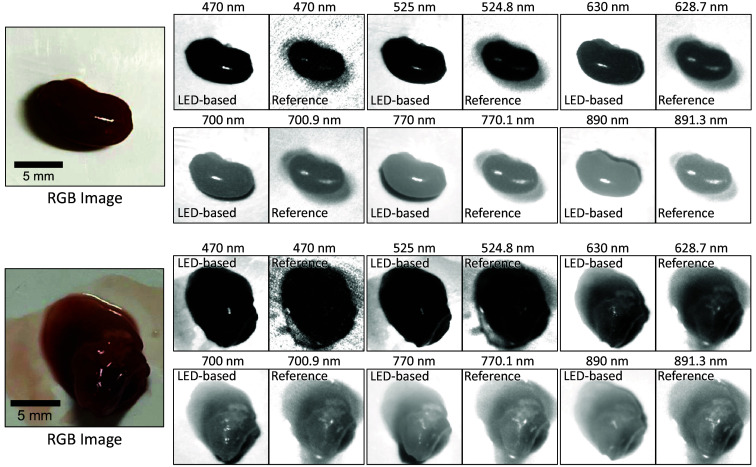
Six sample spectral channels of our LED-based HSI prototype system (left images) and the reference HSI camera (right images) from a normal mouse kidney (top) and a neuroblastoma tumor resected from a mouse kidney (bottom).

**Fig. 8:**
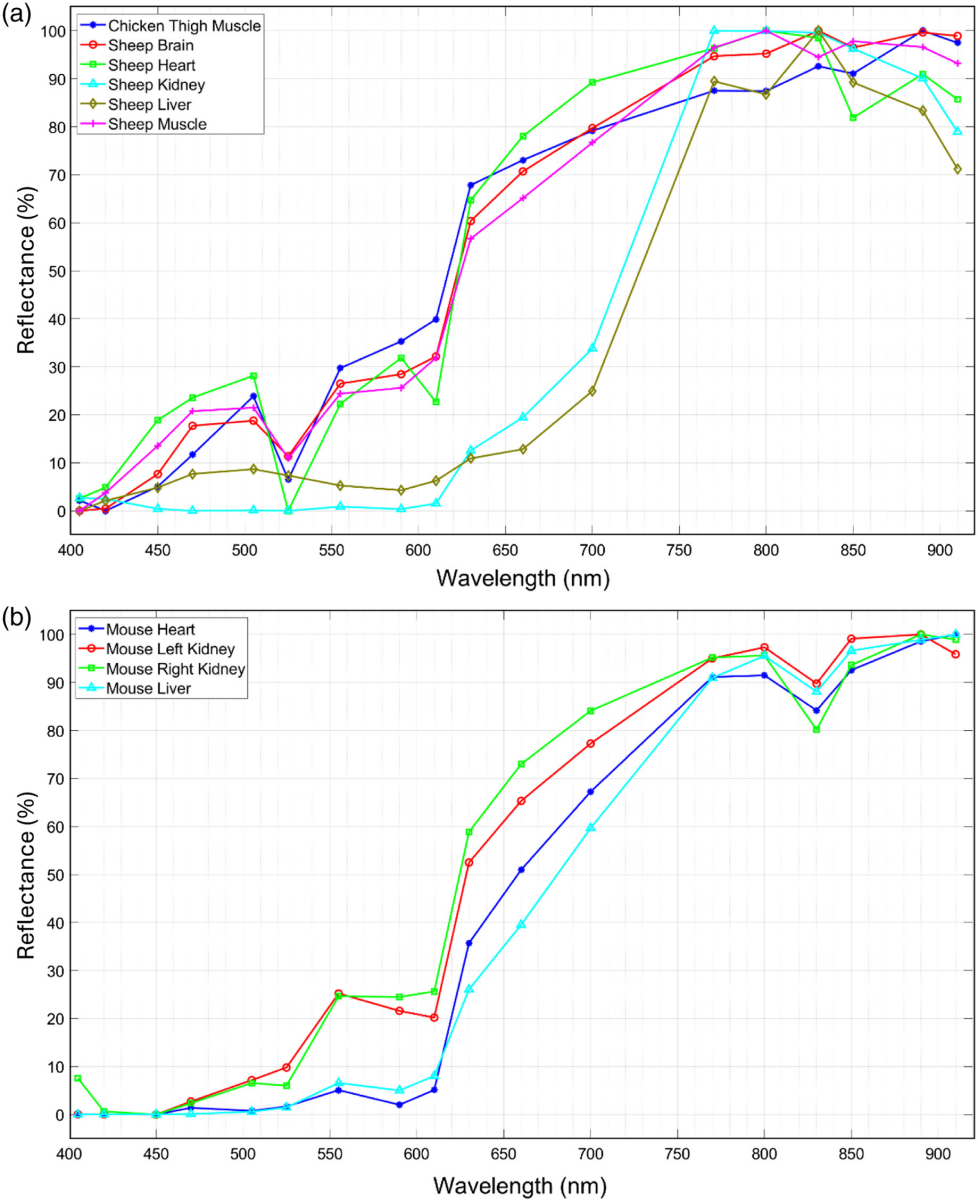
Normalized spectral signatures of *ex vivo* tissues obtained by our LED-based HSI system for: (a) normal tissues resected from sheep and chickens and (b) normal mouse tissues.

**Fig. 9:**
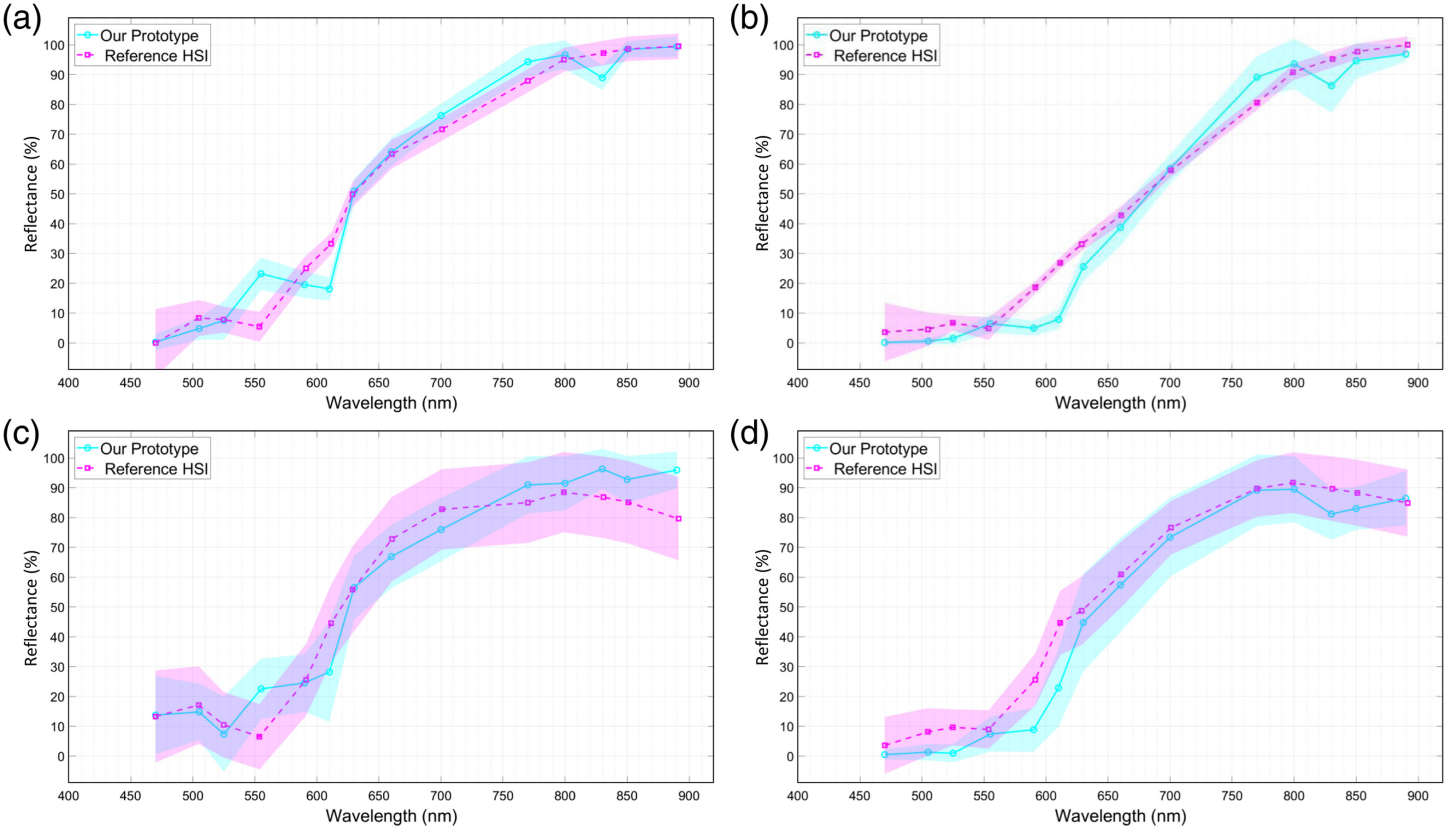
Spectral signature comparison between our LED-based imaging system and the reference camera at the corresponding wavelengths for four sample *ex vivo* tissues: (a) mouse kidney, (b) mouse liver, (c) sheep brain, and (d) neuroblastoma tumor resected from a mouse kidney. The shaded areas represent the standard deviation around the mean values.

**Fig. 10:**
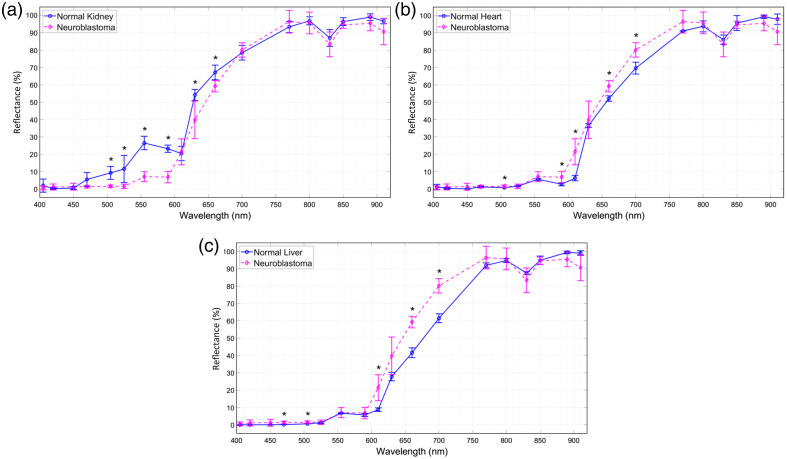
Comparison between the spectral signatures of neuroblastoma tumors in: (a) normal mouse kidneys, (b) normal mouse hearts, and (c) normal mouse livers using our prototype.

Figure 7 shows a set of six sample bands of captured spectral images from two sample *ex vivo* tissues, including a normal mouse kidney and an orthotopically implanted human neuroblastoma tumor resected from a mouse kidney. The figures also show the six corresponding bands from the reference camera hypercubes after image registration. For a better comparison between the two spectral imaging systems, for each tissue, the brightness of the displayed spectral bands was adjusted based on the normalized hyperspectral signature of the tissue. Figure 8 shows the normalized hyperspectral signatures of the normal *ex vivo* tissues from chicken, sheep, and mouse models using our imaging system. Figure 9 compares the normalized hyperspectral signatures of our prototype to those of the reference HSI system for four sample tissues. Figures 9(a) and 9(d), respectively, show the spectral signatures of normal and cancerous tissues shown in Fig. 7. The spectral signatures were plotted at the overlap between the spectral ranges of the two HSI systems and based on the corresponding wavelengths in that range. Figure 10 compares the average spectral signatures of three normal mouse tissues (including the kidney, heart, and liver) to the average signature of neuroblastoma tissue resected from mice using our imaging system. At each spectral band, we compared the normalized reflectivity of the normal tissues to that of the neuroblastoma tumor using student t-tests. We selected one-tailed tests because we assumed that the reflectance values of neuroblastoma tissue would differ from those of normal tissues in a consistent direction (either higher or lower) at certain spectral bands. We used a significant threshold level of 0.05 for our statistical tests. The spectral bands with statistically significant differences in reflectance were indicated with asterisk symbols.

## 4 Discussion

The main features of a hyperspectral endoscopic imaging system are spatial resolution, spectral resolution, FOV, and spectral imaging frame rate. The results of the experiments we conducted in this study showed that the idea of using a spectral LED array combined with a monochrome camera can be used for a high-resolution HSI. Using LEDs as the light source in our imaging system also enables high-speed wavelength scanning HSI. Using micro-LEDs with small footprints enables mounting tens of LEDs at the distal end of a clinical endoscope. This makes it possible to develop a hyperspectral endoscopic imaging system with an *in situ* LED-based illumination source. Using an LED-based wavelength scanning approach makes it a low-power design for endoscopic hyperspectral imaging.

The results of our prototyped system showed a high intra- and inter-spectral band variation in the illumination level and distribution before calibration. The limited illumination angles of the thorough-hole LEDs and the use of one LED per wavelength can be two main factors in that observation. As we used one LED per wavelength, the position and distance of the LEDs from the camera play an important role in the quality of the illumination. In the real-size imaging system, multiple micro-LEDs will be used per wavelength distributed uniformly around the camera lens to improve the homogeneity and illumination intensity for a high-quality imaging. As shown in Fig. 5, the calibration approach we used in this study could compensate for inter- and intra-channel inhomogeneities in illumination when the target surface is flat and normal to the imaging direction. After calibration, the corrected reflectance ratio of all the pixels across the spectrum except for one band (610 nm) is the same at the highest intensity level (100% reflectance), as expected for white reference images. The illumination angle of the LEDs had a high impact on the quality of the images. For wavelengths of 525, 610, and 830 nm, we see a high inhomogeneity of the intensity on the images (specifically at the corners of the images), as shown in Fig. 5. This inhomogeneity was higher by 830 nm with one of the narrowest illumination angles (14 deg) shown in Table 1. As mentioned earlier, this happened because we used through-hole LEDs in our prototype. The lens in the packaging of the through-hole LEDs focuses the light and reduces the illumination angle of the LEDs. For the 610 nm band, the right top corner of the image at that band has been affected by a no-illumination artifact that could not be corrected during the calibration. For those tissues with ROI that was not within the region of the affected corner, the artifact did not affect the hyperspectral signatures. However, the hyperspectral signatures of target tissues within the region of the illumination artifact may be affected at 610 nm. To address this issue in the large-scale prototype, we need to use LEDs with a wider illumination angle (e.g., higher than 60 deg) to avoid imaging artifacts caused by inhomogeneous illumination of the imaged tissue. In a real-size hyperspectral endoscopic imaging model, the illumination angles of the micro-LEDs are wide enough (∼120  deg) to illuminate the entire field of view. Using multiple LEDs per wavelength well distributed in the LED array can also help mitigate the inhomogeneous illumination artifacts.

The qualitative results in Fig. 7 confirm that our system can capture images with quality comparable to our customized HSI camera system. For the bands corresponding to shorter wavelengths (e.g., 470 nm), the imaging quality of our system was even higher than that of the reference camera. However, one reason could be the different sensitivity of the camera sensors to different light wavelengths, which has not been considered in this study.

Figure 8 also shows the capability of our system to differentiate between different *ex vivo* tissues. More specifically, Fig. 10 shows the differences between the hyperspectral signatures of different normal and tumor tissues, with statistically significant differences at five to six bands. We observed statistically significant differences between the healthy mouse kidney, heart, and liver tissues and neuroblastoma tissue at 505 and 660 nm bands. To mitigate the impact of imaging noise on the HSI signatures and the analysis results, the signature of each tissue was plotted based on the average reflectance of the ROI pixels. Figure 9 shows that the hyperspectral signature of our prototype is comparable with that extracted by the reference HSI camera. The angular FOV of the prototyped imaging system was ∼100  deg, which is within the range of FOVs in endoscopic imaging systems.^31^ The hyperspectral imaging rate of 3.3 hps with 18 spectral channels using a monochrome camera with an imaging rate of 120 fps suggests that the proposed approach can be used for real-time endoscopic imaging (imaging rate >10  hps) if a faster camera is used. As the power consumption of the LEDs is low and can be reduced by controlling the forward currents, a low-consumption design can be feasible for applications such as hyperspectral capsule endoscopy.

The materials for the prototype system, including the camera, 60 micro-LEDs, a high-speed wireless transceiver integrated circuit (IC), and connectors, have a cost of less than $1000. With mass production of a commercialized version, the cost can be further reduced. Compared with a regular hyperspectral camera, which could cost tens of thousands of dollars, this LED-based hyperspectral imaging approach can have a significantly lower cost and therefore be applied to low-resource settings.

The observations and results of this study need to be considered in the context of their limitations. In this study, we used a monochrome camera with a maximum 120 fps imaging rate. This limited our tests, as explained before. For real-time hyperspectral imaging with up to 30 spectral channels, we need a camera with a 600 fps imaging rate. The spectral response of the camera sensor also plays an important role in the quality of the imaging. For the camera, we used in this study, the spectrum of the sensor was not available. We removed the IR filter to expand the spectral response of the monochrome camera used in the prototype imaging system. The impact of the spectral characteristics of the camera has been indirectly compensated during the calibration process. In our future study, considering the spectrum of the sensor in our design could be helpful in improving the performance of the imaging system.

Using a set of LEDs with different positions in regard to the imaging target varies the inter-channel shadow and glare pattern. This needs to be addressed in the post-processing steps.

As another limitation of this feasibility study, the LEDs used were through-hole LEDs with different specifications when compared with the micro-LEDs. For the final model, we need to use micro-LEDs and study the quality of the imaging based on their illumination.

The incident angle of the LED array was assumed to be normal in this study. A nonnormal incident angle may affect the quality of illumination and, thereupon, imaging. For example, at a normal imaging angle, the shadowing effect will be minimal. The shadowing could affect the spectral signature of the imaging target. In a nonnormal incident angle, the scattering of the light will be different, which changes the amount of reflected light to the camera. On the other hand, the effectiveness of the calibration process when there are variations in the orientation or surface geometry of the target should be studied in the next steps of this project. It is also important to test the effectiveness of the calibration and the imaging system’s performance in a realistic imaging environment. For example, the hyperspectral signature may change with the angle of the imaging.^32^

The continuous broadband white light of the halogen light sources of the reference imaging system changes the tissue more than the spectral LED-based illumination used in the proposed prototype. This challenges the comparison between the hyperspectral signature of our prototype and the one captured from the reference imaging system. In addition, as we could not image tissue with our prototyped imaging system and the reference camera at the same time, normal drying of the tissue in contact with air could impact the results. This could partially justify the differences observed between the spectral signatures of our prototype and the reference system.

During a clinical endoscopy, the distance between the imaging system and the target is variable. An observation made during our study showed that changing the DOF may affect the captured hyperspectral signature in some bands more than others. Therefore, using an automatic and real-time DOF-related adjustment of the LED illuminations in the final model is required.

For the endoscope, the limited space restricted the number of LEDs that could be used in the LED array light source, and thereupon, the number of spectral channels would be limited. Besides, although the wireless connection improves the mobility of the device, it could become a bottleneck at higher imaging speeds. To transfer all the spectral images in real time, we need high-speed communication between the device and the workstation. For example, using a 400 Mbps data transfer rate enables transferring 10-bit, 30-channel hyperspectral image data in 256×256 size in real time (∼21  hps). However, the higher the data rate, the higher the power consumption of the transceiver IC, which produces heat. Therefore, the data transfer rate needs to be optimized. This affects the image size and the number of spectral channels to be sent in real time.

Although using a battery-powered design facilitates the integration of the device to the distal end of a conventional endoscope, it increases the size of the device, which makes the solid tip of the endoscope longer. It limits the maneuverability of the endoscope. One way to address this issue is to use power lines and remove the batteries. However, this requires major modifications to be applied to the endoscope catheter for the device’s integration into the endoscope catheter. The proposed design could be used for research purposes. In the future, the design could be used for a commercial hyperspectral endoscope that can be used for simultaneous RGB and spectral imaging. The RGB images could be made from the integration of the spectral channels.^33^^,^^34^

## 5 Conclusions

We designed a prototype device for potential hyperspectral endoscopy applications with high quality and low power consumption. The system used a monochrome micro digital camera for capturing the images, which is more cost-effective and smaller than the current hyperspectral imagers. We conducted a feasibility study to characterize the *in situ* LED-based illumination for a wavelength-scanning HSI system. The preliminary results demonstrated that an *in situ* LED-based light source for a hyperspectral endoscope can enable real-time or near-real-time, high-quality, low-noise wavelength-scanning imaging. For future work, we are going to design and implement the imaging module of the hyperspectral endoscope in real size. We will use micro-CMOS LEDs with a footprint size of up to 1 by 1 mm and design a donut-shaped PCB for the LED array with a diameter of up to 12 mm. In addition, we will use a high-speed monochrome camera with a frame rate of around 360 fps to decrease the acquisition time of the hyperspectral endoscope. We will implement the control and processing module on the FPGA for ASIC prototyping, and in the next step, we will design and fabricate an ASIC as a replacement for the FPGA. Using an ASIC helps to decrease the size, power consumption, and cost of the system. This design can also enable the development of hyperspectral capsule endoscopes.

## Data Availability

The datasets generated and analyzed during the current study are not publicly available.
